# New Flexible Silicone-Based EEG Dry Sensor Material Compositions Exhibiting Improvements in Lifespan, Conductivity, and Reliability

**DOI:** 10.3390/s16111826

**Published:** 2016-10-31

**Authors:** Yi-Hsin Yu, Shih-Hsun Chen, Che-Lun Chang, Chin-Teng Lin, W. David Hairston, Randy A. Mrozek

**Affiliations:** 1 Department of Interactive Entertainment Design, China University of Technology, Taipei 11695, Taiwan; ysyou0409@cute.edu.tw; 2 Department of Mechanical Engineering, National Taiwan University of Science and Technology, Taipei 10607, Taiwan; shchen@mail.ntust.edu.tw; 3 Brain Research Center, National Chiao Tung University, Hsinchu 300, Taiwan; chelun@nctu.edu.tw; 4 Faculty of Engineering and Information Technology, University of Technology, Sydney 2007, Australia; 5 Translational Neuroscience Branch, Human Research and Engineering Directorate, U.S. Army Research Laboratory, Adelphi, MD 20783, USA; william.d.hairston4.civ@mail.mil; 6 Macromolecular Science and Technology Branch, Weapons and Materials Research Directorate, U.S. Army Research Laboratory, Adelphi, MD 20783, USA; randy.a.mrozek.civ@mail.mil

**Keywords:** electroencephalography (EEG), scanning electron microscope (SEM), silicone-based dry sensors

## Abstract

This study investigates alternative material compositions for flexible silicone-based dry electroencephalography (EEG) electrodes to improve the performance lifespan while maintaining high-fidelity transmission of EEG signals. Electrode materials were fabricated with varying concentrations of silver-coated silica and silver flakes to evaluate their electrical, mechanical, and EEG transmission performance. Scanning electron microscope (SEM) analysis of the initial electrode development identified some weak points in the sensors’ construction, including particle pull-out and ablation of the silver coating on the silica filler. The newly-developed sensor materials achieved significant improvement in EEG measurements while maintaining the advantages of previous silicone-based electrodes, including flexibility and non-toxicity. The experimental results indicated that the proposed electrodes maintained suitable performance even after exposure to temperature fluctuations, 85% relative humidity, and enhanced corrosion conditions demonstrating improvements in the environmental stability. Fabricated flat (forehead) and acicular (hairy sites) electrodes composed of the optimum identified formulation exhibited low impedance and reliable EEG measurement; some initial human experiments demonstrate the feasibility of using these silicone-based electrodes for typical lab data collection applications.

## 1. Introduction

Bio-signals, such as electrocardiogram (ECG), electroencephalogram (EEG), electrooculogram (EOG), and electromyogram (EMG), are important tools in monitoring people’s physiological conditions for both research and clinical applications [1]. In particular, EEG hardware is cheaper than functional magnetic resonance imaging (fMRI) hardware and also has higher temporal resolution [1,2]. It is also a powerful noninvasive method to reflect the dynamics of brain activities directly [3,4]. In addition to monitoring, EEG can also be applied to real-life warning systems, including driving alertness and sleep therapy [5,6].

Currently, hydrogel-based “wet” EEG electrodes are the most popular option to provide adequate signals and low impedance between the skin and the electrodes. However, using these wet electrodes requires preparation of the skin and direct application of a conductive, water-based gel to obtain suitable EEG performance. In addition to the relatively long and laborious set-up times, the EEG signal may degrade or even disappear as the gel dries due to the relatively high volatility of water at the temperatures experienced by the human scalp [7]. Aside from affecting the electrical properties, drying gel can cause discomfort from abrasion of the outer skin layer and/or allergic reactions [8]. For these reasons, wet electrodes are not an ideal candidate for long-term EEG measurement [9]. As a result, “dry” electrodes are an attractive alternative that may provide easy application, increased comfort, and stable performance to enable long-term EEG measurement.

The most challenging aspect for any EEG electrode is the ability to penetrate the hair in order to make contact with the scalp. Several dry EEG sensors utilize metallic pins to penetrate through the hair and make a mechanical connection with the scalp. Since the sensors must contact the skin directly, the force applied on the sensors may cause discomfort and pain to the user [10,11]. Some dry electrode examples include the g.SAHARA electrode system (the g.tec product) consisting of an eight-pin electrode made of a unique golden alloy [7], spring-loaded EEG dry sensors with several pogo-probes [12], and bristle-sensors with low-cost flexible passive dry EEG electrodes for neurofeedback and brain–computer interface (BCI) applications [13]. Despite these metal pins having a buffer design to reduce the force felt on the scalp by the user, the electrodes are still uncomfortable when worn for an extended period of time [14]. In addition, the metal pins may represent a safety risk if the electrode site were to experience even a low force impact due to the material stiffness. An alternate type of EEG sensor device, microelectromechanical systems (MEMS) electrodes, have been developed for the tips of sensors [15,16] as a way around this because the wires are very small. However, because this kind of sensor is not truly non-invasive, people may have some concerns about using them.

Several critical challenges associated with novel, dry EEG sensor development need to be addressed to enable widespread implementation and adoption by the wider EEG community. These challenges include improved comfort for the wearer, ease of application, the ability to penetrate the hair to provide adequate contact with the scalp, improved durability to provide long performance lifetimes, improved safety outside of a laboratory environment, and consistent performance over long measurement times [17]. Several of these critical challenges must also be addressed to determine whether EEG-based related BCI technology can be accepted by the wider community and, thus, gain wide-spread use [18]. Silicone-based sensors have demonstrated some initial promise in addressing these challenges and have recently passed ”Societe Generale de Surveillance S.A.” (SGS) certifications [14]. For example, flexible dry silicone-based electrodes exhibit very low impedance for transmission of an EEG signal and provide improved safety, comfort, and ease of application relative to several EEG sensor designs previously discussed.

This article investigates the role of the material composition of a conductive polymer composite on the environmental stability under typical EEG conditions and the performance lifetime of dry EEG electrodes. The sensor formulation is composed of a bio-compatible, silicone-based gel along with varying concentrations of one of two silver additives; silver-coated silica spheres (Ag/SiO_2_), silver flakes (Ag-flake). The developed material formulations were evaluated to address three critical performance parameters: (1) determining the optimum composition of each particle to obtain low impedance and high flexibility; (2) determining the influence of filler type and morphology on the performance lifetime of the sensor for long-term applications; and (3) determining the role of the filler morphology and concentration on the performance and durability of the sensor-device interface. The sensors were also examined for environmental stability, deformation-dependent electrical performance, and transmission of an actual EEG signal. These tests were designed to determine the feasibility of using a silicone-based sensor to address the critical challenges limiting the implementation of dry electrodes in EEG systems.

## 2. Materials and Processing

### 2.1. Materials and Processing

The silicone was obtained from Chang Horing Rubber Co., Ltd. (Changhua, Taiwan). Silver-coated silica spheres (12 μm) were obtained from Ecka Granules (Velden, Germany). Silver flakes (13.4 μm) were obtained from Metalor (Neuchâtel, Switzerland). All materials were used as received.

Formulation and casting of the materials was performed by All Position Rubber Enterprise Co., Ltd. (Taipei, Taiwan), using the general protocol of 100 mL batches mixed with a calendaring system at 40 °C temperature. The material was then injection molded and heated to 180 °C for 10 min to obtain complete cure.

### 2.2. Scanning Electron Microscopy Characterization

The samples were coated with an approximately 10 nm thick platinum layer and imaged using a JEOL JSM-6700F scanning electron microscope (JEOL, Peabody, MA, USA) with an accelerating voltage of 15 kV in high vacuum mode.

### 2.3. Environment Testing

Two flat polymer electrode (PE) samples with the best proportion of additives were manufactured first (1: original PE: AgSiO_2_ + silicon; 2: flake PE: Ag flake + silicon). These samples were cut into rectangular samples with dimensions 1 cm × 7 cm × 0.1 cm. The environmental stability of the sensors was evaluated to determine any changes in performance due to temperature, humidity and a salt spray test. The humidity stability was tested under ambient conditions (~30% RH) and high humidity (85% RH) to simulate the boundary conditions of real-world operation. Temperatures of 50 °C and 80 °C were required to maintain the desired relative humidities and, therefore, temperature was also an inherent variable. All tests were held in an air oven for one week, and then the specimens were evaluated by measuring their surface resistivity.

### 2.4. Tensile Testing

**Figure 1:**
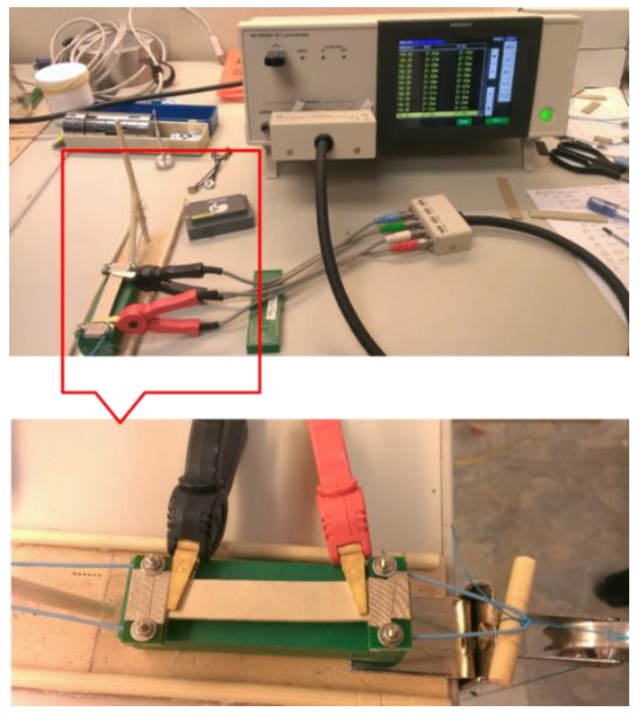
Pictures of the tensile testing apparatus using a pulley along with calibration weights. The impedance was measured by a HIOKI LCR Meter IM3533-01 (HIOKI, Nagano, Japan). Each sample was cut into the pieces 1 cm × 7 cm × 0.1 cm.

In the tensile stress tests, each sample was clamped tightly by a clip at both ends. The first clip was fixed at one end and the second clip was tied to a string through a pulley. The tensile stress was applied by pulling the string with calibration weights, as shown in Figure 1.

### 2.5. Salt Spray Testing

The salt spray test was conducted based on ASTM-B117 standard with saline solutions at three different concentrations, 0.3%, 3.0% and 5.0%, which represented as being very aggressive for a typical environment in an effort to determine any undesirable effects more quickly. The results were also evaluated by their surface resistivity.

### 2.6. Impedance Tests of Dry Electrode and PEs

In general, real-world dry sensors are not self-sufficient; they are usually paired with optimized supporting mechanics and electronics to realize a practical device. Even with acceptable supporting components, a good EEG dry sensor must exhibit low impedance. The Neurosky dry flat electrode (Neurosky, San Jose, CA, USA) is an example of a popular consumer-market EEG sensor. In order to compare the effectiveness of the Neurosky electrode and the proposed electrode, we used a Neurosky device and took apart the electrode. Only impedance tests were conducted to avoid damaging the components.

The impedances of various PEs were also initially recorded, and the changes of impedance as a function of tensile strain were also recorded. Accurate impedance measurements were taken by using an HIOKI IM3523-01 LCR machine (HIOKI, Nagano, Japan). The first clip was fixed on one end and the second clip was tied to the fingers of the electrode.

The EEG signal quality was defined by comparing the correlation values between the flat (forehead) electrode and the conventional wet sensor. In this experiment, the EEG signals were collected using the Neuroscan system (Compumedics Neuroscan, Charlotte, NC, USA). The conventional wet electrode and the flat (forehead) electrode were then placed near the Fp1 location on the subject’s forehead. Next, the difference in the signal quality of the EEG measurements was evaluated using the linear correlation coefficient function in MATLAB (R2007a, The MathWorks, Natick, MA, USA).

### 2.7. Assessment of EEG Data Quality

Upon verifying the signal quality of the electrodes, we wished to ensure analogous function when used on human skin. In order to do so, two types of data were collected. First, as a simple rudimentary test, data from a typical eyes-open, eyes-closed comparison was used to elicit an alpha-burst phenomenon and observe the overall frequency spectrum of the data. Three subjects participated in this test. They wore four electrodes (conventional wet/proposed dry electrodes placed near the Fp1 location on the forehead and wet sensor/modified acicular-PE placed near the Oz location) and sat in a dark experimental chamber. Before the start of the experiment, subjects had three minutes to rest (to enter the resting state). Subjects were instructed to fixate a cross for 20 s and then to keep their eyes closed, also for 20 s. This test acquired 180 s of EEG data, and an eyes-closed sample of the EEG recorded data (20 s) was taken for analysis.

Second, an auditory “oddball” paradigm was used, as this is a common type of application in laboratory experiments. Seven subjects participated in this task to evaluate the functionality of the electrodes. The duration of each task exceeded 10 min. In particular, the auditory P200 topography is a case where a specific component with a typical peak latency of approximately 150–250 ms is elicited by auditory stimuli which do not match a common set [19,20]. In the experiments here, EEG signals from two electrodes were recorded synchronously by the Neuroscan system, with the wet sensor and modified acicular-PE were placed near the Pz location on the parietal region of the subject’s scalp.

## 3. Results and Discussion

### 3.1. Result of Materials Basic Study

**Figure 2:**
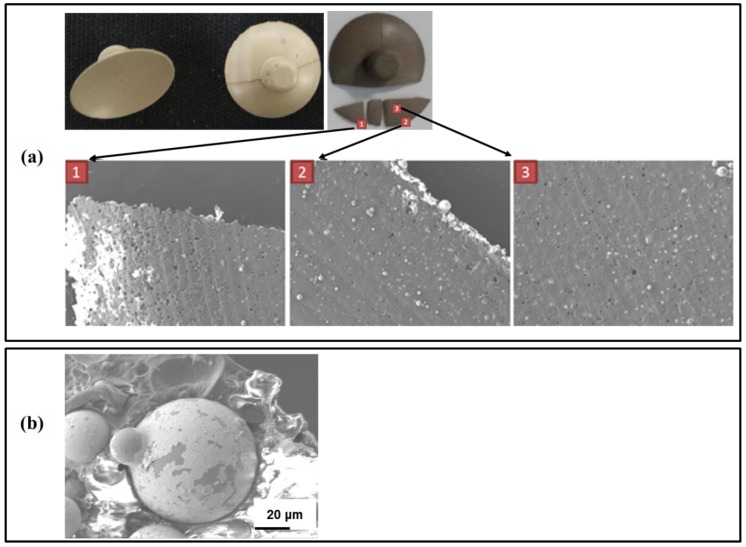
(**a**) Photographs of the top and reverse side of a failed electrode along with SEM images obtained from the labeled locations. The images reveal numerous holes appear on the polymer electrode surface, especially on the edge, after several applications. The nearly spherical holes and the presence of Ag/SiO_2_ particles on the surface seem to indicate particle pull-out; and (**b**) a high-magnification SEM image of the particle surface that seems to indicate that the silver coating is no longer uniform and that the silver is ablated from the silica surface during use.

The feasibility of using polymer electrodes to collect an EEG signal was demonstrated in a previous study [14], however the polymer electrodes did not exhibit reproducible performance over an extended period. After several uses, the conductivity of sensors composed of silver-coated silica (Ag/SiO_2_) was often reduced or lost. In an effort to determine whether any changes were occurring in the electrode microstructure, electrodes that demonstrated a loss of performance were imaged using scanning electron microscopy (SEM) on the top and bottom electrode surface (Figure 2a). The images revealed numerous holes on the polymer electrode surface with the concentration of holes being most prominent on the electrode edge. The holes are almost round in shape consistent with Ag/SiO_2_ particles having been pulled out of the matrix. This observation is further supported by the presence of Ag/SiO_2_ apparently outside the polymer matrix and adhered to the electrode surface. These holes would cause poor electrical contact and reduced conductivity as they are generated. In addition, high magnification images seem to indicate that the silver coating is no longer uniform, which may indicate that the silver is ablated from the silica surface during use (Figure 2b).

Obtaining conductivity in non-conductive silicone requires the addition of conductive particulates at a high enough concentration to form conductive pathways. An important aspect of this study is to determine the useful performance range that provides low impedance while maintaining flexibility and durability. In this study, the concentration of two different particles were evaluated in an effort to reduce the frequency and severity of these failure mechanisms; (1) the original Ag/SiO_2_ and (2) a higher aspect ratio silver flake to reduce the ability for particle pull-out from the silicone matrix. It is worth mentioning that the Ag/SiO_2_ provides significant practical advantages in cost and weight relative to the silver flake; however, silver flake should not exhibit any ablation issues. As summarized in Table 1, several samples with different proportions of additives (Ag/SiO_2_ spheres and Ag flakes) were prepared for testing.

**Figure 3:**
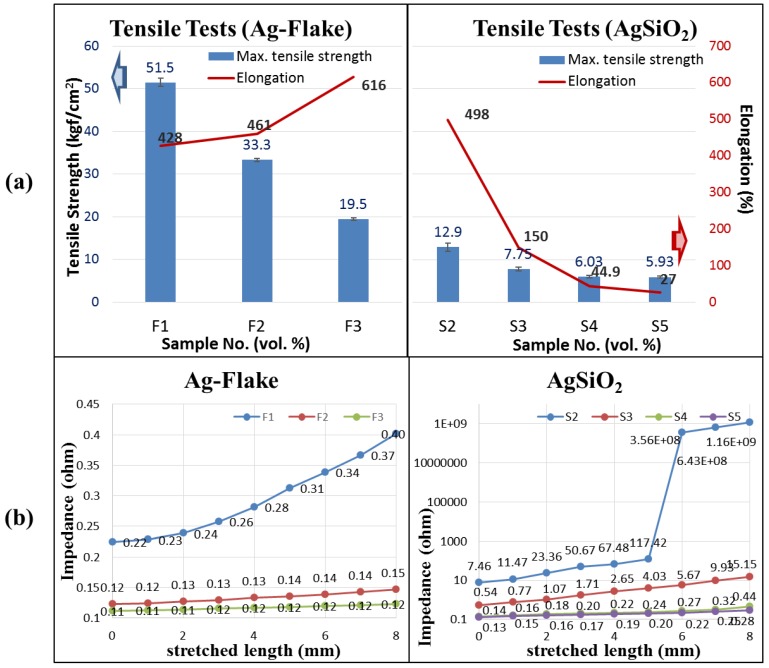
(**a**) Mechanical properties as a function of composition for both Ag-flake and Ag/SiO_2_; (**b**) Strain-dependent impedance measurements obtained from compositions containing Ag-flake and Ag/SiO_2_.

The useful performance range was determined by monitoring the impedance as a function of deformation during a tensile test. Samples composed of Ag-flake concentrations of 35 vol. % and 45 vol. % were not tested because the high Ag-flake content made it impossible to consolidate into a solid sample. Figure 3a shows the results of the tensile tests. The mechanical strength, determined by the elongation at break, of samples reduced while the additive amount increased, consistent with previous literature on particle-loaded elastomeric polymers. Sample elongation was enhanced by Ag-flake additives, but decreased in samples containing Ag/SiO_2_ when the volume percentage of additives was higher than S2 (45%). The conductance of each sample at various strains were also recorded. Figure 3b shows the impedance/stretch results of different proportions of Ag/SiO_2_ and Ag-flake samples. In different proportions of Ag/SiO_2_ analysis, S3, S4, and S5 satisfied the requirement of low impedance. Their impedances did not exceed 20 ohms when the stretched length was about 8 mm [21,22]. However the impedance of S2 was too high to be meaningful when the stretched length was about 6 mm. Samples F2 and F3 exhibited a uniform electrical response even after stretching up to 8 mm, as shown in Figure 2b. Composition F1 exhibited a slight increase in impedance upon elongation, but still maintained sub-ohm impedance values even at an elongation of 8 mm. However, none of the compositions containing silver flake recovered to their original length, which indicates even the relatively low particle concentration in F1 is still enough to reduce the elasticity of the composite material. Material compositions containing AgSiO_2_ above 50 vol. % exhibited minimal changes in resistance with elongation with a maximum impedance value of less than 20 ohms (Figure 3b). According to the above impedance/tension experimental results, we consider the formulation of S3 (volume percentage 1:1) to be a limitative recipe and the formulation is the boundary condition that additives (AgSiO_2_) should not be less than 50%.

### 3.2. Result of Environment Tests for PEs

**Figure 4:**
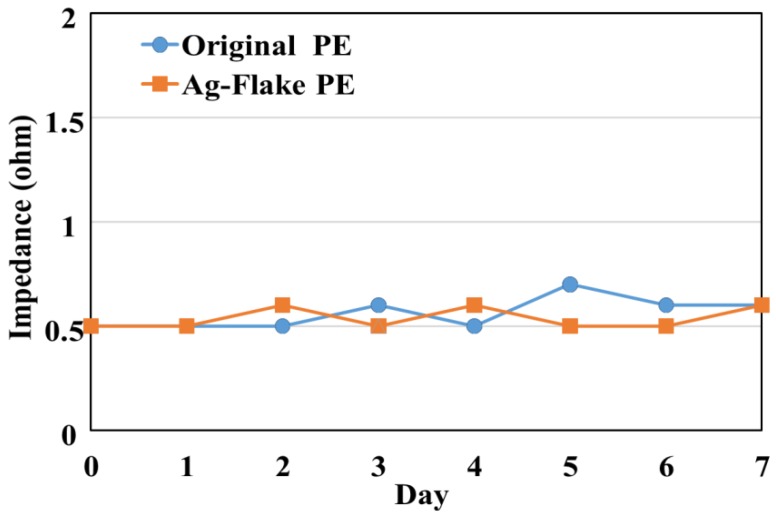
The variation of surface impedance of dry PEs in the temperature and humidity test with 85% RH at 80 °C for one week.

The formulations that exhibited the best combination of mechanical and electrical (original PE combination: silicone:Ag/SiO_2_ = 44%:56%; refined Ag-flake PE combination: silicon:Ag/SiO_2_:Ag-flake = 45%:50%:5%) performance were then evaluated for environmental stability. Maintaining performance under hot and wet conditions is important for long-term EEG utility. Conditions of 80 °C and 85% RH were used as a “worst case” representative environment. Figure 4 shows the sensor impedance as a function of elevated temperature and humidity of various formulations over seven days. After seven days, both of the sensors (original PE (Ag/SiO2) and Ag-flake PE) had maintained their impedance lower than 1 ohm.

**Figure 5:**
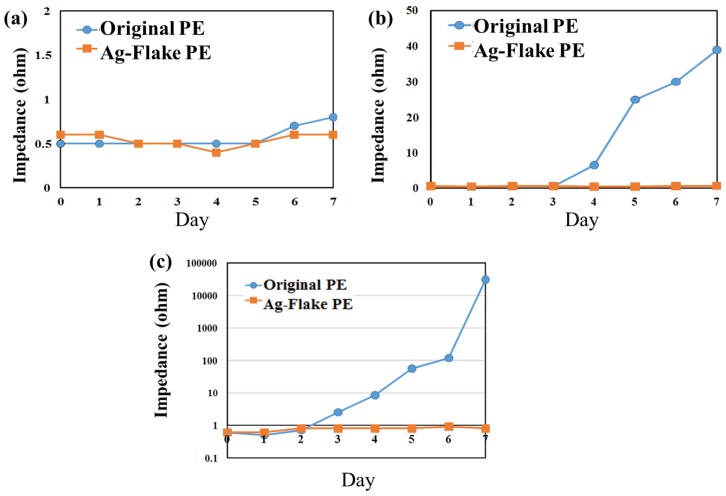
The variations of dry PEs (original PE (Ag/SiO2) and Ag-flake PE) surface impedance after salt spray testing with the saline concentrations of (**a**) 0.3%; (**b**) 3.0%; and (**c**) 5.0% for one week.

The results of the elevated temperature and humidity tests indicated that the dry EEG sensors would maintain their electric properties. However, in practical application, the sensors are subjected to perspiration. The typical saline concentration of human body sweat is about 0.3% (constituents may vary). Figure 5a presents no obvious impedance changes on the 0.3% salt spray test but, at 3.0% concentration, the impedance of the material containing Ag/SiO_2_ started to rise from the fourth day, onwards, and increased by over 40 times after seven-day testing, as shown in Figure 5b. Comparatively, samples containing silver flake were not influenced under the same conditions. In Figure 5c, at 5.0% concentration, the conductivity decay of the sensor composed of Ag/SiO_2_ increased from the second day, onward, and finally reached over 30 (kohms), while there was also no obvious influence on samples containing silver flakes, thus indicating enhanced corrosion resistance relative to Ag/SiO_2_, and which would likely be much less influenced by the human body’s corrosive factors.

### 3.3. Result of Impedance Tests for Neruosky Dry Flat Electrorde and the Proposed Electrode

**Figure 6:**
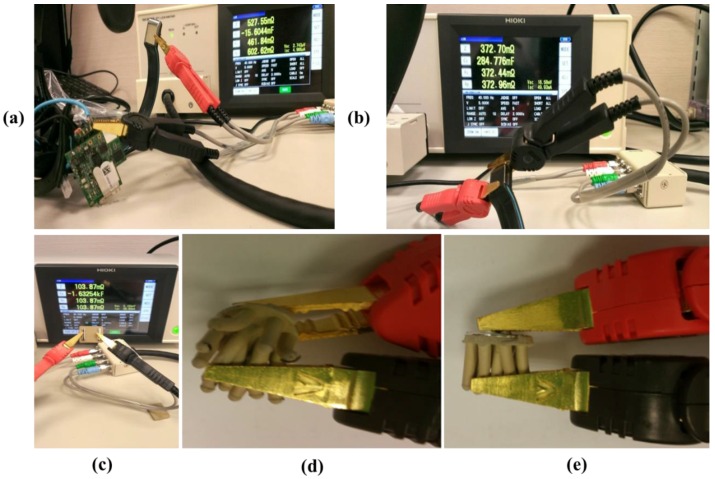
(**a**,**b**) Testing of the impedance of flat Neurosky electrodes; (**c**) Flat silicone-based electrode; (**d**) The bent acicular silicone-based dry sensor; (**e**) The impedance of a normal acicular silicone-based dry sensor.

**Figure 7:**
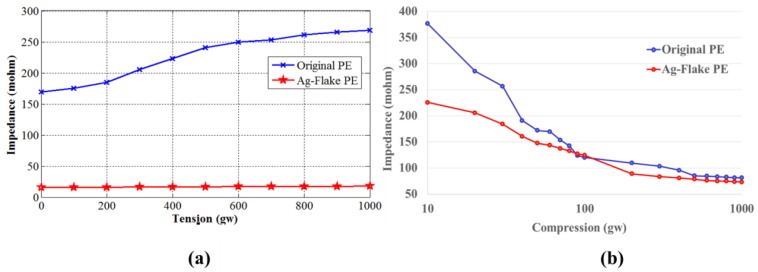
(**a**) Impedance tests of two PEs in different tensile stresses with a frequency of 40 Hz; (**b**) Top-bottom impedance tests of two PEs in different compressions with a frequency of 40 Hz.

Impedance test results are shown in Figure 6. The impedance of flat Neurosky electrodes is about 372 mohms. The impedance of the flat silicone-based dry electrode is lower than 200 mohms, as shown in Figure 7. We conducted several additional tests, and observed (1) the impedance of the flat silicone-based electrode is ~103.9 mohms; (2) the impedance of the bent acicular silicone-based dry sensor is ~39.4 mohms; and (3) the impedance of normal acicular silicone-based dry sensor is ~38.65 mohms.

### 3.4. Result of Impedance Tests under Different Tensile Stresses and Compressions

The strain-dependent impedance was determined in both tensile and compression geometry. Figure 6 shows impedance tests of two PEs (original PE (Ag/SiO_2_) and Ag-flake PE) in different tension and compression conditions. The input frequency of tests was set at 40 Hz (benchmark to general EEG systems, such as the NeuroScan system). Figure 7a shows the impedances increased with the weights. The impedance of flake is lower than the original one. Figure 7b shows the top-bottom impedance changes under different compressions. The impedance changes of the sample containing silver flakes were less than the others. In general, this indicated that, in both tensile and compression tests, the compositions containing silver flakes had lower impedances than those containing Ag/SiO_2_, like the original PE. In real life situations, it is most likely that EEG electrodes will be deformed in tension or compression to provide adequate scalp contact.

### 3.5. Physiological Potentials and EEG Signal Tests

**Figure 8:**
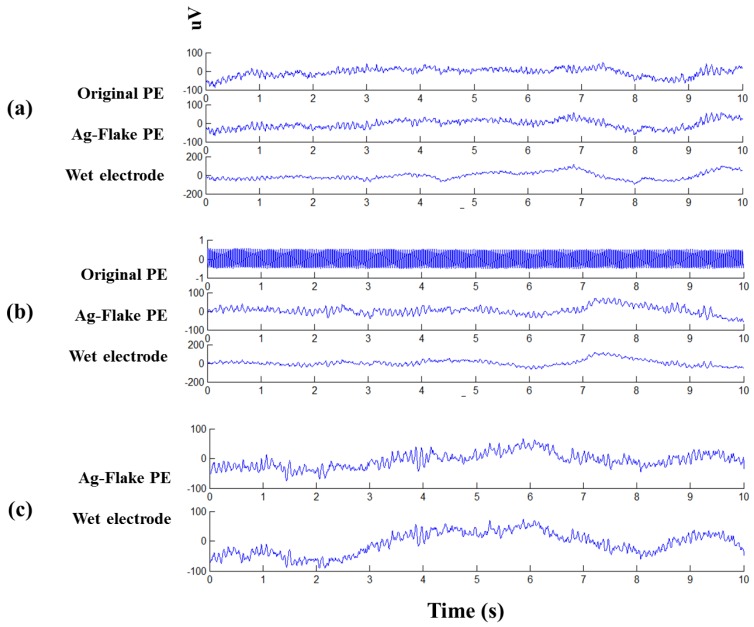
Synchronic EEG tracings of eyes closed conditions for different electrodes (original PE, Ag-flake PE, and wet electrode) underwent the salt spray test in (**a**) 0.3%; (**b**) 3.0%; and (**c**) 5.0% saline solution.

EEG sensors were fabricated using the identified formulations to determine the initial feasibility of implementation into EEG systems. In the following tests, two dry PEs (the original electrode and the refined Ag-flake electrode) and conventional wet electrodes were utilized to measure eyes-closed EEG signals. Of note is that the PEs tested had already undergone salt spray tests in saline solutions of 0.3%, 3.0%, and 5.0%. In Figure 8a, typical eyes-closed EEG signals were clearly observed across all electrodes. Additionally, alpha rhythms were synchronously presented on all interfaces [9,23,24]. Notably, data from the original electrode exposed to a 3.0% saline solution was invalid, as shown in Figure 8b. This clearly demonstrates the importance of maintaining low impedance in these materials for use as EEG sensors. Consistent with the salt spray testing, the improved electrode maintained an accurate response even after exposure to 5.0% salinity, as shown in Figure 8c.

### 3.6. Modified EEG Sensors Prototype Signal Tests

**Figure 9:**
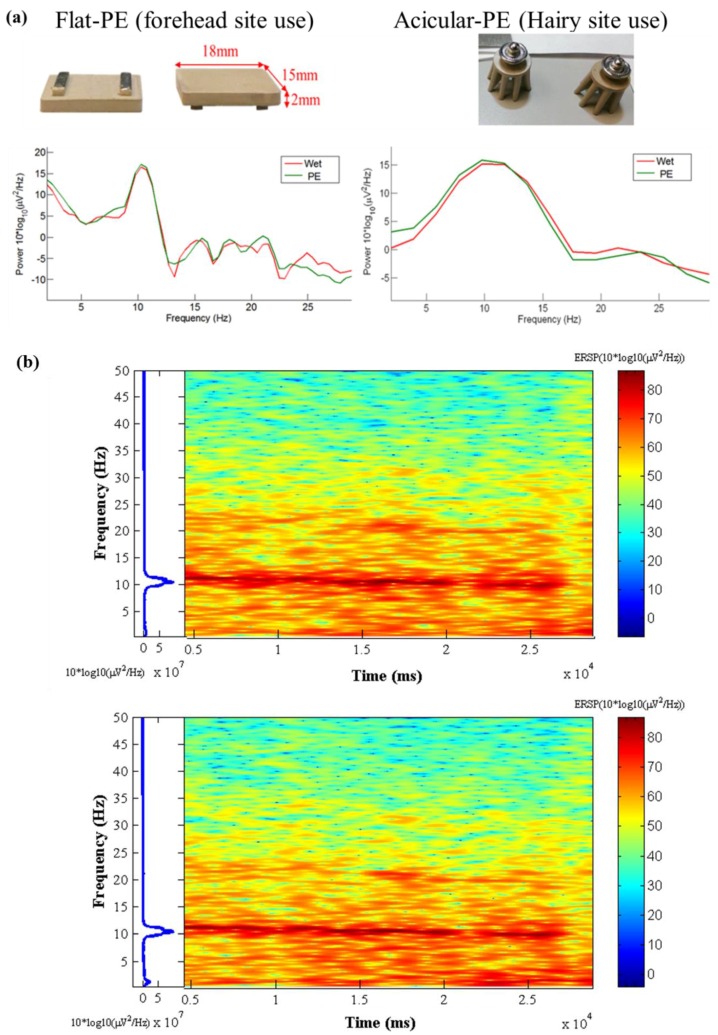
(**a**) Alpha rhythm tests. The left diagram is the forehead EEG data and the right is the occipital EEG data. Wet sensors were located at Fp1and Oz and the proposed dry PEs were located near Fp1 and Oz; (**b**) Time-frequency analysis of the EEG signals (Oz) for 0~50 Hz spectrum. The top diagram is the wet sensor’s result located at Oz, and the bottom diagram is the proposed PE’s result located near Oz.

**Figure 10:**
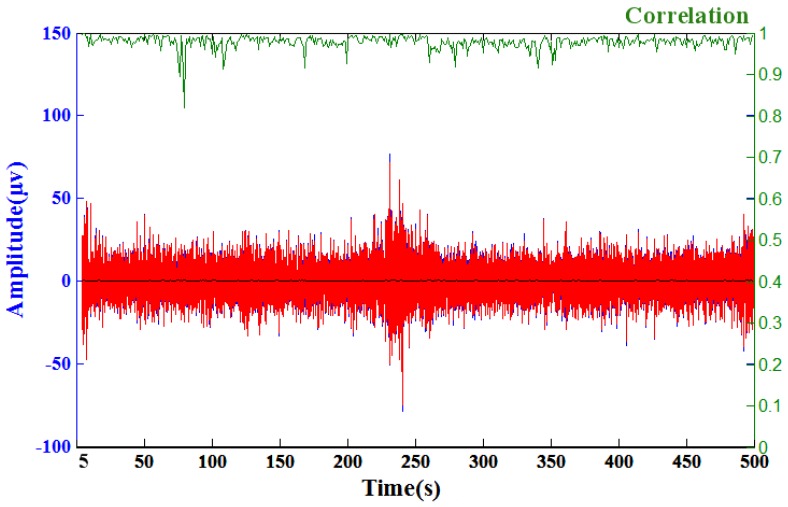
A comparison of the EEG signals recorded by a wet electrode (red curve) and the acicular Ag-flake PE (blue curve). The green curve shows the correlation of these two signals over a sliding one-second window and the black curve shows the root mean square error (RMSE) of these two signals. The average correlation between the blue and red lines is high (~97.85%), the maximum of the RMSE is 0.69 (μv) and the average of RMSE is 0.13 (μv). This test acquired 700 s of EEG data, and a sample of the EEG recorded data (5~500 s) was taken for analysis.

After achieving favorable performances of sensors containing silver flakes, prototypes of EEG sensors were manufactured and tested. A flat sensors were used for forehead site EEG measurements and acicular sensors were used for hairy site EEG measurements. The flat sensors had embedded metal contact bars and the acicular sensors had an embedded metal male button for easy connection to the EEG acquisition devices. Typical alpha rhythm tests [9,23,24] were conducted. Corresponding Ag-flake PE sensors and wet sensors with gel were located at the frontal (FP1) and occipital (Oz) position nearby [25,26]. Figure 9a shows the resting state of the EEG signals during an example 20 s eyes-closed window for wet and PE sensors. The left diagram is the forehead EEG data and the right is the occipital EEG data. Wet sensors were located at Fp1 and Oz, and proposed dry PEs were located near Fp1 and Oz. The alpha phenomena were observed in both EEG signals for the wet sensor (red curves) and proposed PEs (green curves). Figure 9b shows the time-frequency analysis of the EEG signals (Oz) for the 0~50 Hz spectrum. The top diagram is the wet sensor’s result located at Oz, the bottom diagram is the proposed PE’s result located near Oz. Moreover, the EEG signal correlation analysis between the Ag-flake PE and the wet electrode revealed a correlation averaging 97.85%. The wet sensor with gel and the proposed dry sensor were located at positions near Pz. Figure 10 shows the related correlation analysis and statistics results.

### 3.7. EEG Oddball Experiment for Quality Verification

**Figure 11:**
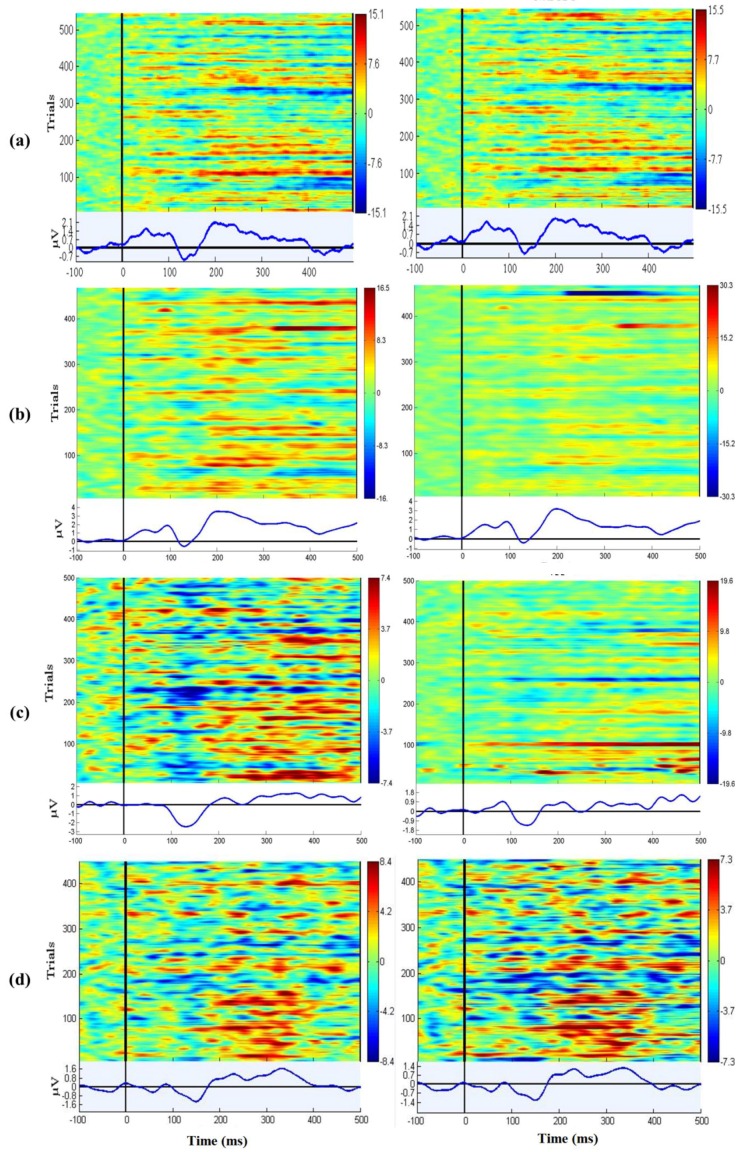

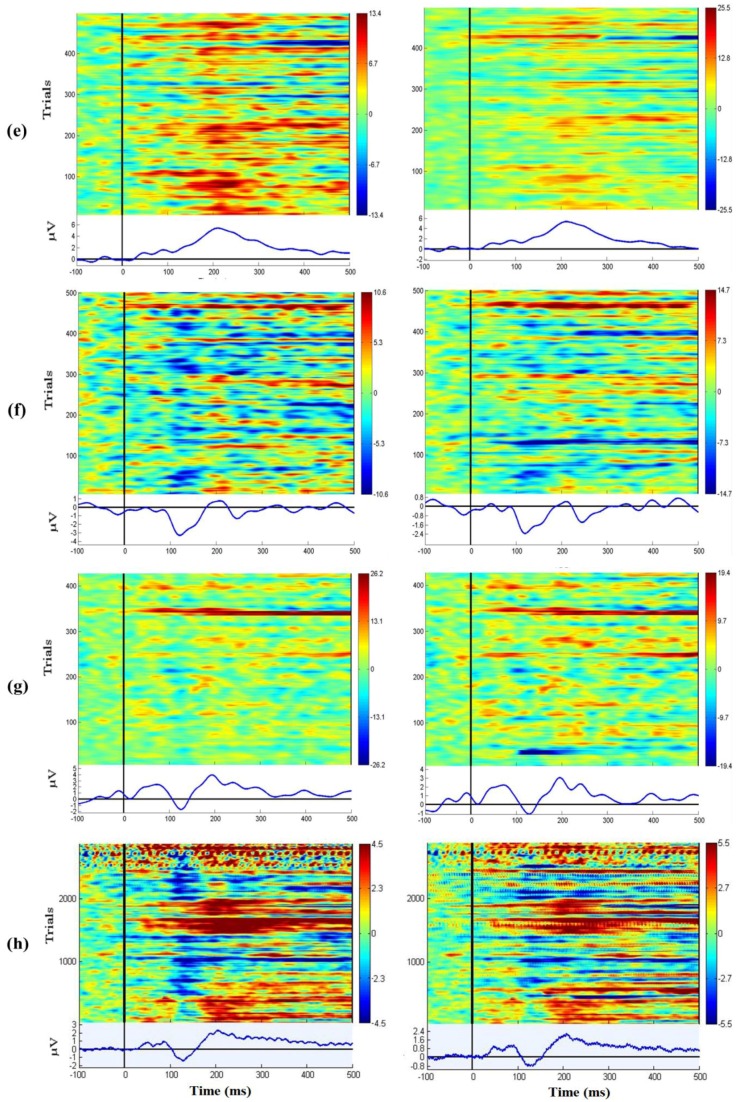
(**a**–**g**) The P200 phenomena were presented in the ERPs for both wet and dry channels for each subject; (**h**) shows the grand mean P200 components were detected by the subjects’ EEG data.

Oddball tasks are common EEG experiments which are used to verify whether the EEG signal quality is sufficient [14,27]. EEGLAB (v10.2.5.5b, Swartz Center for Computational Neuroscience, San Diego, CA, USA) and MATLAB were used to evaluate the event-related potential (ERP) maps [28,29]. Seven subjects participated in this task, which lasted for a duration of at least 10 min. The P200 component occurs when there is a positive detection, and has a typical peak latency of approximately 150–250 ms elicited by auditory stimuli [19,20]. For the Figure 11a–g, the left parts of the figure are each subject’s wet sensors’ ERP and the right parts of the figure are each subject’s proposed sensors’ ERP. Figure 11h shows the P200 components were detected by the subjects’ EEG data. While there is clear variability in the overall response between subjects, there is a clear similarity and high correlation in the variance across time and trials within each subject when comparing the wet (left side) and dry polymer (right side) data.

## 4. Conclusions

Silicone-based composites containing two different particle types, silver-coated silica and silver flake at varying concentration were evaluated for potential use as EEG sensors. This work determined that a useful performance range could be achieved that provided a suitable combination of electrical and mechanical performance with both particle types. Both of the materials exhibited good temperature and humidity uniformity; however, materials composed of silver-coated silica were determined to be susceptible to changes in impedance upon exposure to a saline solution. As a result, materials composed of silver flake are likely more suitable for practical application where perspiration is expected.

The material exhibited only minor changes in electrical performance upon deformation in tensile and compressive strain. Sample electrodes of the respective materials demonstrated feasibility for high-quality recording of EEG signals. Experimental results (using the Neuroscan system, a conventional, off-the-shelf device) showed that the proposed dry polymer electrodes can provide lower contact impedance and high anti-corrosion ability. Without the requirements of skin preparation and conducting gel, the dry polymer electrodes can provide lower and stable impedance for long-term EEG measurements. The EEG signal qualities achieved by the proposed dry polymer electrodes were coherent to that of wet electrodes. Experimental results show the proposed dry polymer electrodes sensors would not only overcome the drawbacks of wet electrodes but also have good performance in EEG measurements. Since silicone elastomers and silver are both biocompatible substances, the proposed dry polymer electrodes will be a good candidate for EEG measurements in research and clinical study.

## Figures and Tables

**Table 1:** The list of several samples of different proportions of additives (Ag/SiO_2_ and Ag-flake).

	Ag/SiO_2_			Ag-Flake		
	(~2.75 g/mL)			(10.5 g/mL)		
	Volume Percentage			Volume Percentage		
	Sample No.	Silicone	Ag/SiO_2_	Sample No.	Silicone	Ag-Flake
**More**	**S5**	**40%**	**60%**	**F5**	**55%**	**45%**
**↑**	**S4**	**44%**	**56%**	**F4**	**65%**	**35%**
**Additives**			**(origin)**			
**↓**	**S3**	**50%**	**50%**	**F3**	**75%**	**25%**
**Less**	**S2**	**55%**	**45%**	**F2**	**85%**	**15%**
	**S1**	**60%**	**40%**	**F1**	**95%**	**5%**

## Supplementary Materials

The following are available online at http://www.mdpi.com/1424-8220/16/11/1826/s1.
